# The Complete Sequence of a West Nile Virus Lineage 2 Strain Detected in a *Hyalomma marginatum marginatum* Tick Collected from a Song Thrush (*Turdus philomelos*) in Eastern Romania in 2013 Revealed Closest Genetic Relationship to Strain Volgograd 2007

**DOI:** 10.1371/journal.pone.0109905

**Published:** 2014-10-03

**Authors:** Jolanta Kolodziejek, Mihai Marinov, Botond J. Kiss, Vasile Alexe, Norbert Nowotny

**Affiliations:** 1 Viral Zoonoses, Emerging and Vector-Borne Infections Group, Institute of Virology, University of Veterinary Medicine Vienna, Vienna, Austria; 2 Danube Delta National Institute for Research and Development, Tulcea, Romania; 3 Department of Microbiology and Immunology, College of Medicine and Health Sciences, Sultan Qaboos University, Muscat, Oman; University of California Davis, United States of America

## Abstract

In this study the first complete sequence of the West Nile virus (WNV) lineage 2 strain currently circulating in Romania was determined. The virus was detected in a *Hyalomma marginatum marginatum* tick collected from a juvenile song thrush (*Turdus philomelos*) in the Romanian Danube Delta close to the city of Tulcea, end of August 2013. Our finding emphasizes the role of ticks in introduction and maintenance of WNV infections. Sequence analyses revealed close genetic relationship of the Romanian WNV strain to strain Reb_Volgograd_07_H, which was isolated from human brain tissue during an outbreak of West Nile neuroinvasive disease (WNND) in Russia in 2007. In 2010 the Eastern European lineage 2 WNV caused an outbreak of human WNND in Romania. Partial sequences from subsequent years demonstrated that this WNV strain became endemic in Eastern Europe and has been causing outbreaks of varying sizes in southern Russia since 2007 and in Romania since 2010.

## Introduction

Mosquitoes, primarily of the genus *Culex*, are considered the main vectors of West Nile virus (WNV), a zoonotic member of the genus *Flavivirus*. Wild birds constitute the principal hosts of the virus amplifying it in a bird-mosquito cycle. Certain ‘bridge’ mosquito species have been determined to transmit the virus to humans and other mammals, which are regarded dead-end hosts [1], [2]. The role of ticks as WNV vectors had been poorly investigated to date [3].

Romania has a long-standing history of WNV infections, including severe outbreaks of human West Nile neuroinvasive disease (WNND) in 1996 with 393 confirmed cases [4], and in 2010 with 57 cases. Affected patients were distributed among 19 districts in the southern, western, central and eastern parts of the country [5]. The ‘2010’ WNV strain became endemic and has been the cause of outbreaks of varying sizes each following year (http://www.ecdc.europa.eu/en/healthtopics/west_nile_fever/Pages/epidemiological_updates.aspx; http://www.ecdc.europa.eu/en/healthtopics/west_nile_fever/West-Nile-fever-maps/Pages/historical-data.aspx). Nevertheless, the complete sequence of the main WNV strain circulating in Romania since 2010 has not been determined as yet. Therefore goals of this study were to determine the complete genomic sequence of the WNV strain currently circulating in Romania, assess its pathogenicity and neuroinvasive markers, investigate its phylogenetic relatedness to other WNV strains, and discuss the role of ticks in WNV introduction and maintenance.

## Materials and Methods

A total of 32 ticks were found randomly on a total of 23 birds, which had been captured using mist nets [6] in the Danube Delta Biosphere Reserve, Romania. They were investigated for the presence of WNV within the framework of the European Union FP7 project EDENext. Specifically, the birds were captured at the following locations: Enisala (44°53′28.28″N/28°49′50.97″E), Gr. Lupilor (44°41′46.82″N/28°56′15.70″E), Salcioara (44°47′55.30″N/28°53′57.57″E), Maliuc Mila (45°10′34.61″N/29° 3′53.91″E), Rachitarie (45°11′34.74″N/29° 5′8.16″E), and Maliuc (45°10′35.96″N/29° 6′29.51″E). All ethical and research certifications, approvals and permits were issued by the responsible authorities, i.e. the Romanian Ornithological Centre, Danube Delta Biosphere Reserve Authority, Sanitary, Veterinary and Food Safety County Direction Tulcea, and the Scientific Council of the Danube Delta National Institute. The field studies did not involve endangered or protected species. The tick-infested birds were *Passer montanus* (n = 20), *Acrocephalus arundinaceus* (1), *Turdus philomelos* (1) and *Oriolus oriolus* (1). The latter was found to be most infested by ticks (n = 10), whereas merely a single tick was retrieved from each of the remaining birds. Ticks from *Acrocephalus arundinaceus* and *Oriolus oriolus* (n = 11) were collected in May 2012, from four *Passer montanus* in July 2012, and from sixteen further *Passer montanus* as well as one *Turdus philomelos* in August 2013. The ticks were removed using tweezers and stored at −80°C until further investigation.

Ticks were homogenized individually in a TissueLyser II (Qiagen, USA) using Tungsten Carbide Beads 3 mm (Qiagen, USA) and 150 µl nuclease free water (Promega, Madison, USA) for 3 min at 30 Hz. Both genomic DNA and total RNA were extracted from each suspension employing the QIAamp Viral RNA Mini Kit (Qiagen, USA) following the manufacturer's instructions.

For rapid detection of both WNV lineages 1 and 2 in the samples, a reliable real-time RT-qPCR method [WNV (lin.1+2) RT-qPCR] targeting the highly conserved 5′ non-coding region (NCR) was established. The sequences were as follows: WNV_8F: 5′-CGCCTGTGTGAGCTGACAAA-3′, WNV_118R 5′-GCCCTCCTGGTTTCTTAGACATC-3′, and WNV_67T: 5′-FAM-TGCGAGCTGTTTCTTAGCACGA-TAMRA-3′ for the forward primer, reverse primer, and TaqMan probe, respectively. The numbering refers to the WNV lineage 1 sequence AF196835.

Primers and probe for the WNV (lin.1+2) RT-qPCR were designed with the help of the AbiPrism Primer Express 2.0.0 software (Applied Biosystems, USA). The real-time RT-qPCR was performed in an Applied Biosystems 7300 Real-Time PCR System using a SuperScript III Platinum One-Step Quantitative RT-PCR System kit (Invitrogen, USA) following the manufacturer's instructions.

The positive sample was subsequently tested by various RT-PCRs using published universal flavivirus primer pairs, e.g., [7]–[9], primers specific for WNV lineage 2 [10] (online appendix: http://www.cdc.gov/ncidod/EID/vol12no04/05-1379_app.htm], and self-designed primers (Table 1)).

**Table 1:** In this table primer pairs which have been used in addition to primers described by Bakonyi et al. [10] are listed (available as online appendix at: http://www.cdc.gov/ncidod/EID/vol12no04/05-1379_app.htm).

Sequence 5′–3′ (F, forward primer; R, reverse primer)	Primer position (refers to the sequence VLG_07, FJ425721)	Length of the PCR product (nt)
AGCACGAAGATCTCGATGTC(F)*	49–68	593
GTGCACCARCAGTCRATGTC(R)*	641–622	
CCGCGGATTGTCCTTGATAG(F)	129–148	766
CACGACGCGTTGCATYGTGT(R)	894–875	
CAATCTGTTGTGGCTCTAGG(F)	1681–1700	845
TCCATCCAGGCTTCCACATC(R)	2525–2506	
GCCGGAGCGATTCCTGTTGA(F)	1732–1751	1340
AGCTTCCARGTGTCGTTGAG(R)	3071–3052	
TCCTTGCAGTTGGAGGAGTT(F)	2387–2406	826
CCTGGTCTCCTGTTGTGATT(R)	3212–3193	
GGCACGCACAACCACTGAGA(F)	3327–3346	642
AGCAGCGGCACCACCACATT(R)	3968–3949	
GGCCTGCTACAGAAGTGATG(F)	4190–4209	552
CCTTAGTGGTGTGCCACAGT(R)	4741–4722	
GGTCTGGCAGAACTTGACAT(F)	4243–4262	431
CCAAGCAGACCTCGAGTCAT(R)	4673–4654	
TGCTGAGATCACAGGCTCTA(F)	4380–4399	1021
GTGTGGAGACATCAGCCTAT(R)	5400–5381	
AGATTGAGGACGGCTGTGCT(F)	5224–5243	638
TTGCGGCTGTCGATCACTCT(R)	5861–5842	
ATAGGCTGATGTCTCCACAC(F)	5381–5400	591
TTCTTCCTATGCGTCCTCT(R)	5971–5953	
CAGAGGCTCGCATCATGCTA(F)	6047–6066	795
ACACAGCGAGCTGGTTGTCA(R)	6841–6822	
CCTGAGCGCGAGAAGGTGTA(F)	6112–6131	828
GTGTCCTAGCAGGCTGCTAA(R)	6939–6920	
CCTGAGAACAGCTGACTTAC(F)	6189–6208	519
GTGGCAGCTCCTAAGATTAC(R)	6707–6688	
TGTTGGATGGCTGAAGTCTC(F)	6715–6734	789
TGCTGCTGCTGTAGTCAGAA(R)	7503–7484	
GACTCTGACCGTGACTGTGA(F)	7203–7222	715
ATAGCACCAGCCGCCTCTAC(R)	7917–7898	
AGCGGAAGCTATGCGATCTG(F)	7275–7294	847
TTCTACCTCGGCACTTGACG(R)	8121–8102	
GGCCATTACTGAAGTTGACC(F)	7740–7759	1061
ACAGCCAGTTCGTGGTCTCA(R)	8800–8781	
ACCGTCCGTGTCTTGGAGAT(F)	8131–8150	448
TTCCGTGGTAGTTCCAGGT(R)	8578–8560	
AGCTGACCTCGAGAATGAAG(F)	9288–9307	990
CGGACCTGATTRATTGCTAC(R)	10277–10258	
TAGCGCGGTCCATCATCGAG(F)	9348–9367	1633
GCGCACTGTGCCGTGTGGCT(R)	10980–10961	
GCCACCGGAAGTTGAGTAGA(F)*	10515–10534	466
CTGGTTGTGCAGAGCAGAG(R)*	10962–10944	
GCTGCGAGGTGATCCACGTA(F)	10579–10598	398
ACTGTGCCGTGTGGCTGGTT(R)	10976–10957	

Primers marked with stars are suitable for detection of both WNV lineages 1 and 2.

All primer pairs for the conventional RT-PCRs were developed with the help of Primer Designer program (Scientific & Educational Software). The RT-PCR assays were carried out using One Step RT-PCR Kit (Qiagen, USA).

Primer and probe synthesis as well as sequencing in both directions were carried out by Microsynth (Balgach, Switzerland).

The obtained WNV sequences were verified by BLAST search and compiled to one continuous sequence. The complete genome of the newly determined WNV was compared with 23 other WNV strains, representing complete lineage 2 sequences from different hosts, countries and years. Multiple alignments were performed using BioEdit Sequence Alignment Editor (version 7.0.9.0) and verified by Clustal X program (version 1.8).

Phylogenetic neighbor joining analysis was conducted with the help of the MEGA5 program. The evolutionary distances were computed using Maximum Composite Likelihood model [11]. Bootstrap resampling analysis with 1000 replicates was employed. Information about several sequences deposited atGenBank was obtained from [12]. Sequence translation was carried out using the program (http://www.expasy.org/genomics).

To explore the pathogenicity and neuroinvasiveness markers of the newly determined WNV strain, predicted N-glycosylation sites of all viral proteins were analyzed using the program NetNGlyc 1.0 (http://www.cbs.dtu.dk/services/NetNGlyc/) according to [13] (summarized in [14]).

In order to explore the hypothesis of the existence of a single Eastern European lineage 2 WNV cluster, partial E and partial NS5 gene sequences from Russia and Romania, available in GenBank (Table 2) were phylogenetically analyzed as described above for the complete genome sequences. Unfortunately the Russian and Romanian partial sequences targeted different WNV genes, thus additional two phylogenetic trees had to be established. Due to various lengths, the sequences had to be adjusted to 474 nt (E gene sequences) and 466 nt (NS5 gene sequences), respectively.

**Table 2:** Characteristics of WNV lineage 2 partial sequences additionally included in Figures 2 and 3.

GenBank acc. no.	Designation	Length (nt)	Country	Area	Collection date	Host	Reference
**Partial WNV E gene sequences additionally included in ** **Figure 2**							
FJ265699	Isolate VLG-07-2657	1563	Russia	Volgograd	2007	Human (blood)	Platonov et al., GenBank
FJ265700	Isolate VLG-07-26428	1563	Russia	Volgograd	2007	Human (blood)	Platonov et al., GenBank
FJ265701	Isolate VLG-07-26571	1563	Russia	Volgograd	2007	Human (blood)	Platonov et al., GenBank
FJ265702	Isolate VLG-07-26298	1563	Russia	Volgograd	2007	Human (blood)	Platonov et al., GenBank
FJ265703	Isolate VLG-07-26304	1563	Russia	Volgograd	2007	Human (blood)	Platonov et al., GenBank
FJ425729	Isolate 57_VLG_07_M	1563	Russia	Volgograd	2007	Mosquito (*Culex pipiens*)	Platonov et al., GenBank
HQ237494	Isolate ROS-2-2010-H	774	Russia	Rostov	2010	Human (blood)	Karan et al., GenBank
HQ237498	Isolate VLG-609-2010-H	774	Russia	Volgograd	2010	Human (brain)	Karan et al., Genbank
JQ014116	Isolate VOLGOGRAD-01/918-2011	474 (length-limiting sequence)	Russia	Volgograd	2011	Human (urine)	Antonov et al., GenBank
JX844662	Isolate VOLGOGRAD-03/619-2012	492	Russia	Volgograd	2012	Human (brain)	Antonov et al., GenBank
**Partial WNV NS5 gene sequences additionally included in ** **Figure 3**							
HE984574	Isolate 89-2011	1189 (with 100nt gap in the middle)	Romania	Danube Delta (Mila 26)	2011	Mosquito (*Culex pipiens*)	Panculescu-Gatej et al., GenBank
HG328830	Isolate RO_mo151/2012	642	Romania	Not available	07-Sep-2012	Mosquito (*Culex pipiens*)	Dinu et al., Genbank
HG328831	Isolate RO_hu121351/2012	705	Romania	Not available	2012	Human (serum)	Dinu et al., GenBank
HG514461	Isolate RO_mo48/2012	618	Romania	Danube Delta (Mila 26)	26-Aug-2012	Mosquito (*Culex pipiens*)	Dinu et al., GenBank
HG514462	Isolate RO_mo98/2012	636	Romania	Danube Delta (Mila 26)	25-Aug-2012	Mosquito (*Culex pipiens*)	Dinu et al., Genbank
HG514463	Isolate RO_mo418/2012	614	Romania	Danube Delta (Mila 26)	30-Sep-2012	Mosquito (*Culex modestus*)	Dinu et al., Genbank
HG514464	Isolate RO_mo419-2012	585 (length-limiting sequence)	Romania	Danube Delta (Mila 26)	30-Sep-2012	Mosquito (*Culex modestus*)	Dinu et al., Genbank
HG514465	Isolate RO_mo426/2012	633	Romania	Danube Delta (Mila 26)	30-Sep-2012	Mosquito (*Culex modestus*)	Dinu et al., GenBank
HG514466	Isolate RO_mo434-2012	638	Romania	Danube Delta (Mila 26)	29-Sep-2012	Mosquito (*Anopheles hyrcanus*)	Dinu et al., GenBank
HG514467	Isolate RO_mo444-2012	633	Romania	Danube Delta (Mila 26)	30-Sep-2012	Mosquito (*Culex pipiens*)	Dinu et al., GenBank
HG514468	Isolate RO_mo460/2012	639	Romania	Danube Delta (Mila 26)	28-Sep-2012	Mosquito (*Culex pipiens*)	Dinu et al., GenBank
LK022077	Isolate RO_mo292-2013	795	Romania	Danube Delta (Mila 26)	04-Aug-2013	Mosquito (*Culex pipiens*)	Dinu et al., GenBank
LK022078	Isolate RO_mo294/2013	664	Romania	Danube Delta (Mila 26)	04-Aug-2013	Mosquito (*Culex pipiens*)	Dinu et al., GenBank
LK022079	Isolate RO_mo313-2013	752	Romania	Danube Delta (Mila 26)	05-Aug-2013	Mosquito (*Culex pipiens*)	Dinu et al., GenBank
LK022080	Isolate RO_mo354/2013	673	Romania	Danube Delta (Mila 26)	02-Sep-2013	Mosquito (*Culex pipiens*)	Dinu et al., GenBank
LK022081	1isolate RO_mo531/2013	728	Romania	Bucharest	25-Aug-2013	Mosquito (*Culex pipiens*)	Dinu et al., GenBank
HG918026	Isolate RO_hu149704/2013	600	Romania	Bucharest	02-Sep-2013	Human (serum)	Dinu et al., GenBank
HG918027	Isolate RO_hu149802/2013	562 (length-limiting sequence)	Romania	Fetesti	02-Sep-2013	Human (serum)	Dinu et al., GenBank
HG918029	Isolate RO_mo10/2013	705	Romania	Danube Delta (Mila 26)	28-Aug-2013	Mosquito (*Coquillettidia richiardii*)	Dinu et al., GenBank
HG918031	Isolate RO_mo17/2013	681	Romania	Danube Delta (Mila 26)	28-Aug-2013	Mosquito (*Culex pipiens*)	Dinu et al., GenBank
HG918033	Isolate RO_mo34/2013	666	Romania	Danube Delta (Mila 26)	28-Aug-2013	Mosquito (*Coquillettidia richiardii*)	Dinu et al., GenBank
HG918036	Isolate RO_mo168/2013	614	Romania	Danube Delta (Mila 26)	02-Sep-2013	Mosquito (*Culex pipiens*)	Dinu et al., GenBank
HG918037	Isolate RO_mo233-2013	582	Romania	Danube Delta (Mila 26)	01-Aug-2013	Mosquito (*Culex pipiens*)	Dinu et al., GenBank

Molecular determination of the tick species was performed using a PCR assay targeting the mitochondrial 12S rDNA gene with primers recommended previously [15], [16]. For this purpose a Fast Cycling PCR Kit (Qiagen, USA) was applied.

Upon removal of the tick from the bird, oral and cloacal swabs as well as serum were collected from the bird, and later tested using the above-mentioned real-time WNV (lin.1+2) RT-qPCR. The serum sample was additionally investigated for the presence of antibodies against WNV by INGESIM West Nile Compac ELISA (Ingenasa, Madrid, Spain) following the manufacturer's instructions, and by PRNT [17], [18].

## Results

One immature tick (nymph) was positive upon WNV (lin.1+2) RT-qPCR. This tick was genetically identified as *Hyalomma marginatun marginatum* (*H. m. marginatum*). It was found on a juvenile song thrush (*Turdus philomelos*) which had been captured in a mist-net in Enisala/Romanian Danube Delta on 27.08.2013. All other investigated ticks tested negative. All WNV-negative ticks were also identified as *H. m. marginatum*, except one tick which was identified as *Haemaphyalis* sp. (collected in August 2013 from a *Passer montanus*).

By application of several published and self-designed primer pairs a complete, 11,013 nt long WNV sequence was generated from the infected tick, encoding a 3,434 aa long polyprotein, which consists of all typical WNV proteins C, prM, M, E, NS1, NS2a, NS2b, NS3, NS4a, NS4b and NS5 with the corresponding lengths of 123, 92, 75, 501, 352, 231, 131, 619, 149, 256 and 905 aa, respectively.

The Romanian tick-derived WNV was most closely related to strain Reb_VLG_07_H (GenBank acc.no. FJ425721) with only 58 nucleotide differences (identity rate 99.45%).

The comparison of the polyprotein sequences of both WNV strains revealed only six amino acid substitutions (identity rate 99.83%): Thr to Ile (position 108 of the C gene), Ser to Gly (position 199 of the E gene), Met to Leu (pos. 90 of the NS2a gene), Ser to Pro (pos. 100 of the NS4a gene), as well as Tyr to His and Ala to Glu (positions 18 and 370 of the NS5 gene), of which two amino acid substitutions at positions 108 in C gene and 199 in E gene were unique for the Romanian tick WNV, compared to all complete WNV genomes investigated in this study.

The known pathogenicity and neuroinvasiveness markers could be identified in the Romanian WNV: N-glycosylation motif NYS at position 154 of the E protein as well as three potential N-glycosylation sites at positions 130, 175, and 207 in the NS1 gene, prolin at position 250 of the NS1 gene and histidine at position 249 of the NS3 gene.

**Figure 1:**
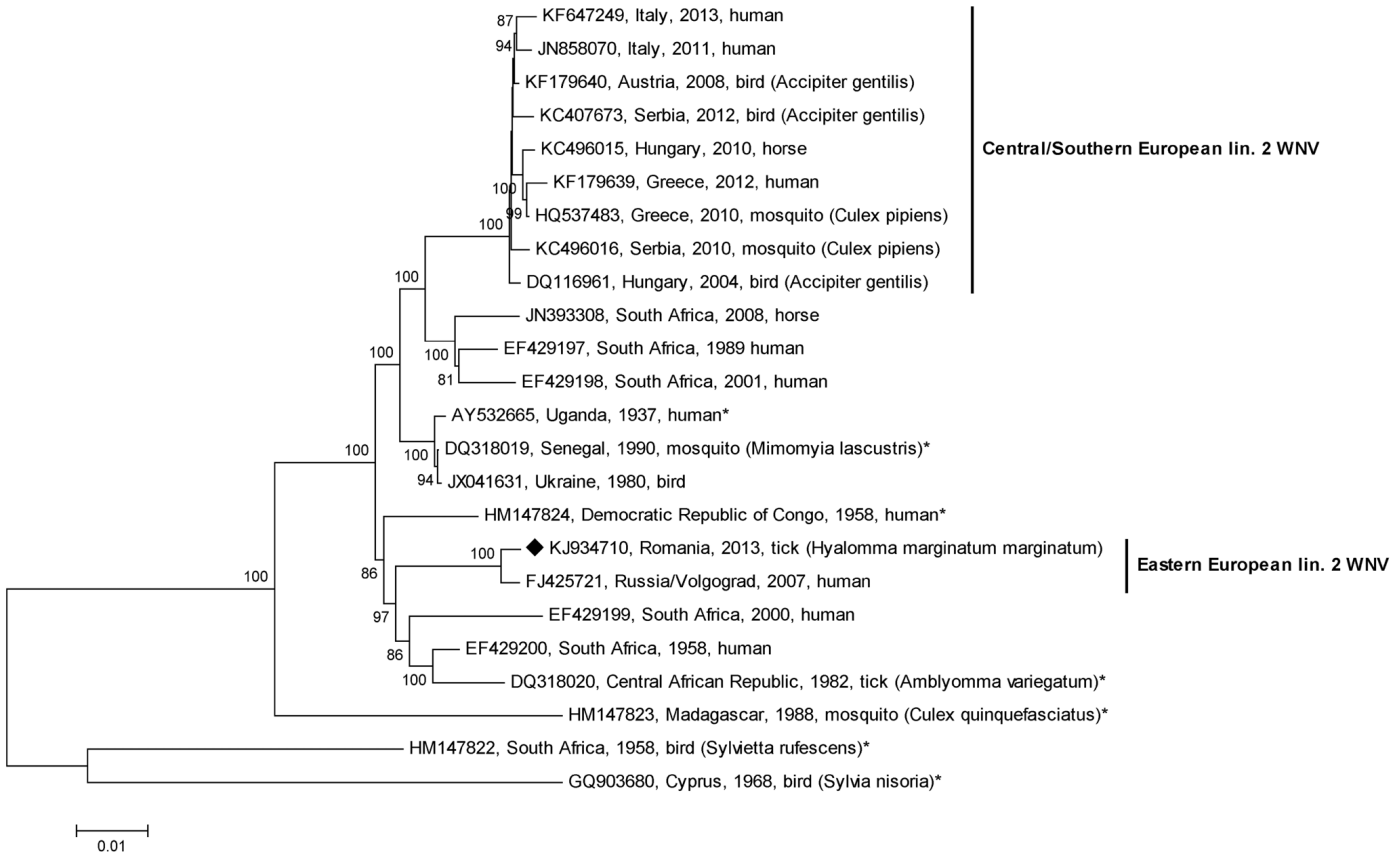
Phylogenetic tree of 24 representative WNV lineage 2 complete genomic sequences. The WNV sequence derived from a *Hyalomma marginatum marginatum* tick collected from a song thrush in Romania (marked with a black diamond) is most closely related to the human-derived WNV strain VLG_07 from Russia. All other WNV strains related to this Russian/Romanian cluster originate from Central and South Africa, suggesting an introduction of this WNV lineage 2 variant from Africa to Europe. The cluster of another independent introduction of a WNV lineage 2 to Central Europe is also indicated. Black stars indicate sequences for which information was obtained from McMullen et al. [12]. The percentage of replicates in the bootstrap test (1000 replicates) is shown next to the branches. Values less than 70% are hidden.

Phylogenetic analysis of 24 complete WNV lineage 2 sequences confirmed the close genetic relationship of the newly determined Romanian tick WNV with the Russian human-derived WNV from 2007 (Figure 1). All other viruses in this major cluster are of African origin, while the Central/Southern European lineage 2 viruses form an independent clade (Figure 1).

Comparison of the WNV strain obtained in this study with partial human and mosquito-derived E gene sequences of 10 Russian WNVs obtained between 2007 and 2012 as well as with partial human- and mosquito-derived NS5 gene sequences of 23 Romanian WNVs obtained between 2011 and 2013 exhibited both 99–100% nucleotide identities.

**Figure 2:**
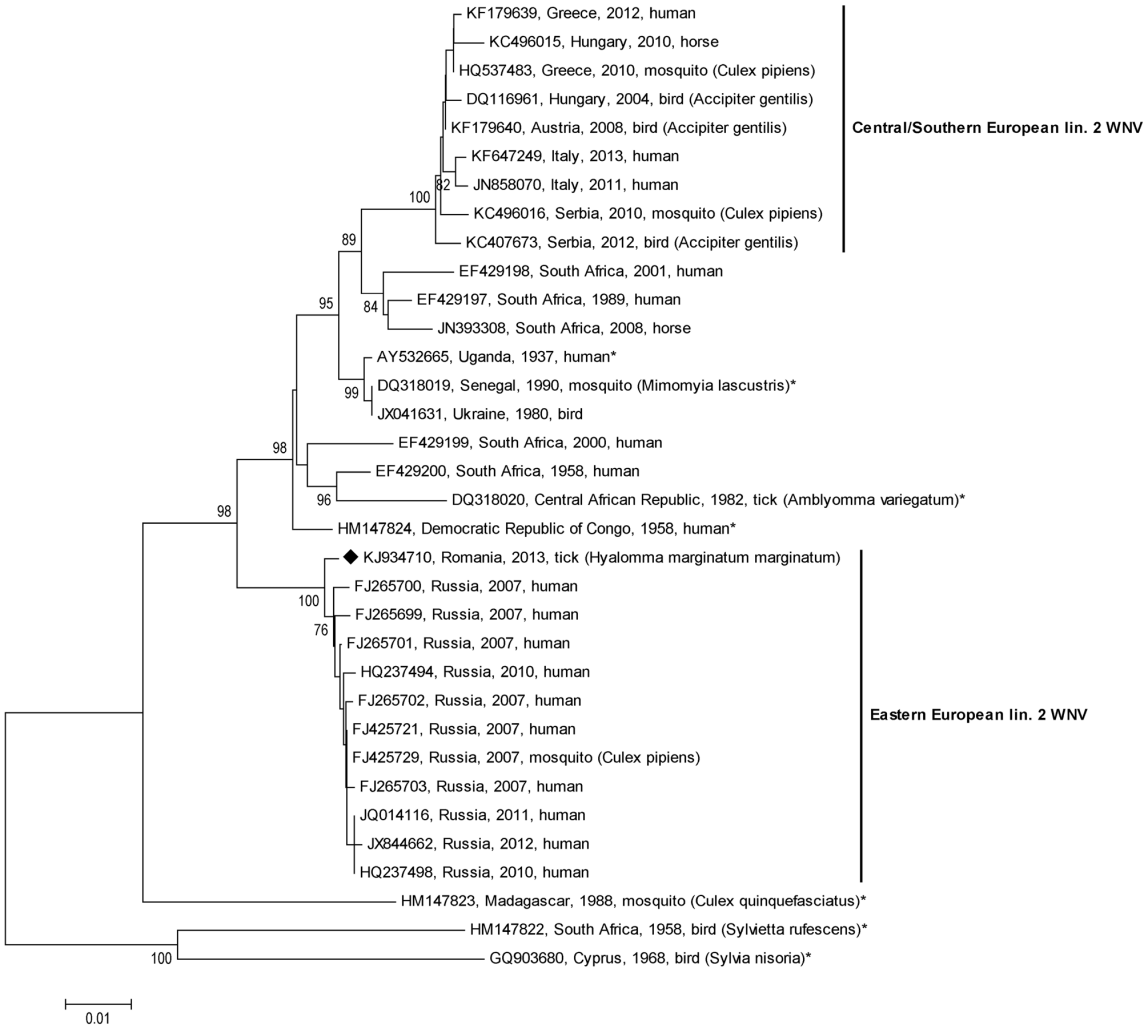
Phylogenetic tree of 474 nt long nucleic acid sequences (corresponding to nucleotide positions 1002–1475 of reference strain Reb_VLG_07_H, GenBank acc. no. FJ425721) within the E gene of the 24 WNV sequences included in **Figure 1** and additional 10 WNVs isolated in Russia between 2007 and 2013. Please note the distinct Eastern European lineage 2 WNV cluster consisting of Russian and Romanian sequences.

**Figure 3:**
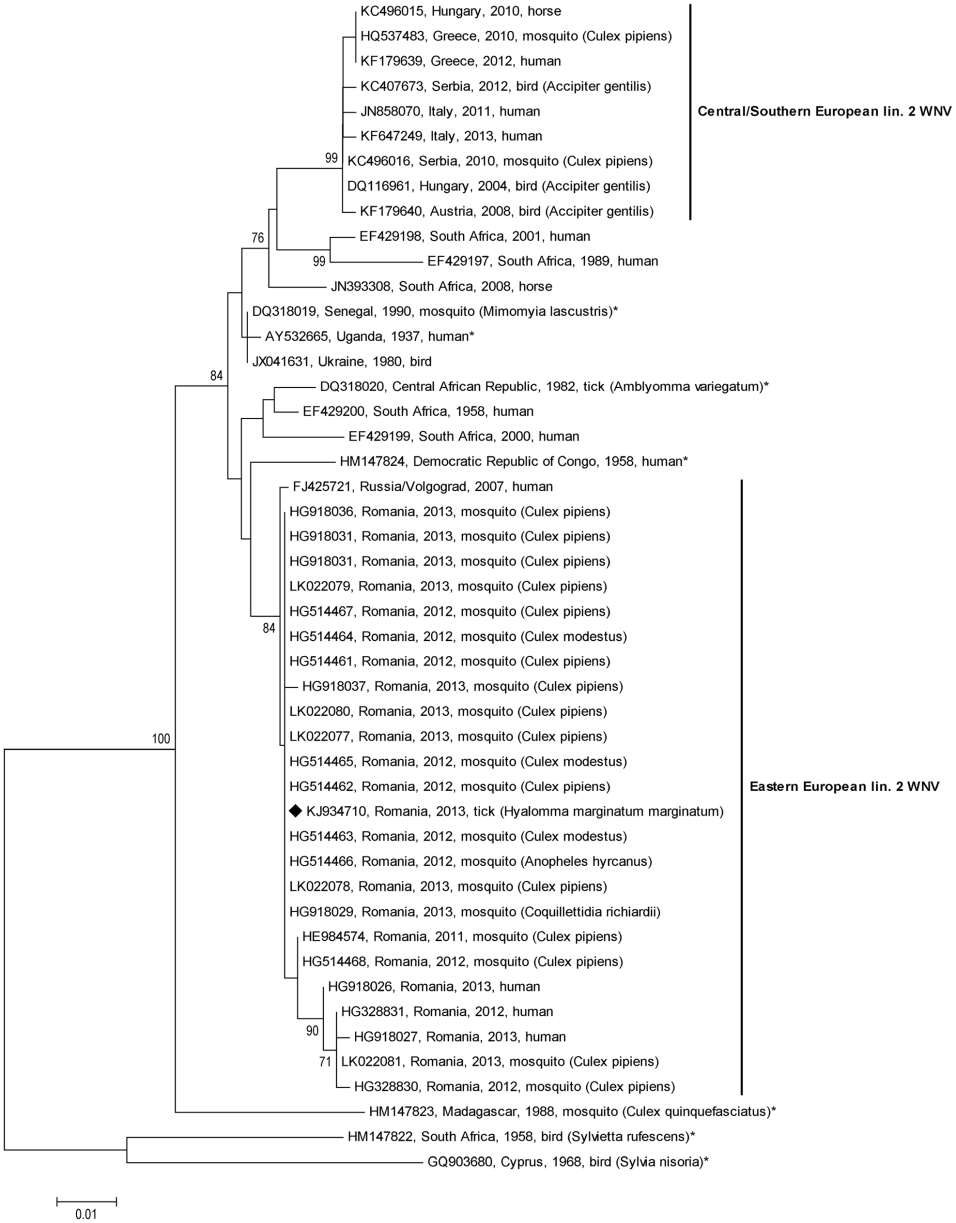
Phylogenetic tree of 466 nt long nucleic acid sequences (corresponding to nucleotide positions 9463–9928 of reference strain Reb_VLG_07_H, GenBank acc. no. FJ425721) within the NS5 gene of the 24 WNV sequences included in **Figure 1** and additional 23 WNVs identified in Romania between 2011 and 2013. Please note the distinct Eastern European lineage 2 WNV cluster consisting of Romanian and Russian sequences.

The phylogenetic analyses of the partial E gene sequences (Figure 2) and NS5 gene sequences (Figure 3), respectively, revealed a single Eastern European lineage 2 WNV cluster of closely related Russian and Romanian sequences from 2007 to 2013.

Nucleic acid extracts of both cloacal and oral swabs of the bird which carried the WNV positive tick as well as of its serum were negative upon the WNV (lin.1+2) RT-qPCR. The serum of this bird tested weakly-positive by WNV antibody ELISA. The confirmatory PRNT assay revealed a borderline positive result.

The complete sequence of the newly determined WNV strain, termed WNV lineage 2 strain Hyalomma/Romania/2013, is available from GenBank under accession number KJ934710. The 341bp long 12S rDNA gene sequence of the WNV-positive *Hyalomma* tick is available at GenBank under accession number KJ862057.

## Discussion

The first major human WNV epidemic in Europe occurred in Romania in 1996, with a high rate of neurological symptoms [4]. A WNV lineage 1 was subsequently determined as the cause of this outbreak, and introduction of this virus by migrating birds from sub-Saharan Africa was suggested [19]. Interestingly, a closely related WNV was the etiologic agent of another large outbreak of WNND in 1999 in the Volgograd region of Russia with more than 800 hospitalized patients [20].

A decade later, a similar sequence of events was noticed, this time, however, the Volgograd outbreak (2007 [21]) occurred three years prior to the Romanian outbreak (2010 [5]). A newly introduced WNV lineage 2 strain was responsible for both outbreaks. The very close genetic relationship of the Romanian virus with the Russian Reb_Volgograd_07_H virus has been demonstrated in the present paper. Sirbu et al. [5] already reported that a 780 nt long WNV sequence determined from serum of an affected patient was 99.3% identical to strain Volgograd_07. In the following years several Romanian partial WNV sequences within the NS5 gene, obtained from patients and mosquitoes, were submitted to GenBank (Table 2). Our align analyses confirmed their close relationships to the above-mentioned Russian strain and revealed almost 100% nucleotide identity with the *Hyalomma*-derived strain determined in this study. Partial E gene sequences of Russian WNVs isolated between 2007 and 2012 (Table 2) confirmed the close relationship between Russian and Romanian lineage 2 WNV strains, too, and phylogenetic analyses of both partial E (Figure 2) and NS5 (Figure 3) gene sequences resulted in a distinct Russian/Romanian WNV lineage 2 genetic cluster, indicating local circulation and persistence of this WNV cluster in Eastern Europe.

The genetically most similar relatives of the Russian/Romanian lineage 2 WNV cluster are much older African viruses (Figures 1–3), suggesting an introduction of this virus from Africa, possibly via migrating birds. Interestingly, one of these old African WNV strains was isolated from a tick [12].

A slightly earlier independent introduction of a WNV lineage 2 from Africa to Europe occurred in or before 2004 [10]. This virus strain also managed toprevail and spread from Central Europe [22] via the Balkan states [23] to Southern European countries such as Greece [24] and Italy [25].

Ciccozzi et al. [26] suggested that the WNV lineage 2 introduction to Central Europe took place around 1999, followed by an independent introduction of another lineage 2 strain to Russia in the year 2000.

Bucharest and Volgograd are approximately 1,500 km apart, however, several flightpaths of certain species of birds, e.g. the song thrush, between these regions exist. It was not possible to determine whether the captured song thrush was a migrating or local bird. As a migrant, the song thrush breeds in most of Europe, and its migration to the Mediterranean starts in late August [27]. Its journey crosses Romania.

Migrating birds have been generally accepted as vehicles carrying viruses from Africa to Europe. Frequently, however, viremia lasts for merely a week in birds [2], a period which is considered too short to introduce exotic viruses to Europe.

Ticks are known carriers of viruses. In Israel, a total of 1.6% of *Argas arboreus* tick pools collected from wild and domestic birds and their nests proved WNV-positive, however all *Hyalomma* species tested negative [28]. WNV RNA and antigen were also detected in the tick species *Ixodes pavlovskyi and I. persulcatus*, which were collected from small mammals, lizards and birds in the region of Tomsk, Russia, at an average rate between 5.2 and 11.7% [29]. Depending on the tick species, WNV may persist for a very long time in ticks, e.g. at least 132 days, as demonstrated by [3].

Experimental infection with WNV performed on four ixodid tick species in the USA [30] and on *H. marginatum* ticks in Portugal [31] revealed that these tick species were able to acquire the virus from infected animals and to transmit it between various developmental stages. In case of *H. marginatum* nymphs and adults, subsequent virus transmission to uninfected hosts was observed [31]. *H. marginatum* is a ‘hard tick species’ occurring in southern and eastern Europe, South Asia and Africa. It is a common ectoparasite of – especially passerine – birds. Immature *Hyalomma* ticks may remain attached to their vertebrate hosts for up to four weeks, which enables their passive transport across continents (http://www.ecdc.europa.eu/en/healthtopics/vectors/ticks/Pages/hyalomma-marginatum-.aspx). As a two-host species moulting from larva to nymph on its first host and infesting the second host as an adult, *H. marginatum* ticks are able to infest a broad spectrum of vertebrate hosts including birds [32]–[34] and humans [35], [36], thereby disseminating WNV infection. Although *H. marginatum* usually prefers relatively dry and warm regions with low humidity, its import to Germany [37], the Netherlands [38], the United Kingdom [32], and Russia [39] has already been reported.

In the present study, the song thrush had cleared WNV, as evidenced by absence of viral RNA in samples of the bird and a low WNV antibody titer. In the attached tick, however, WNV persisted.

In the current study one out of 32 investigated ticks proved to be infected with WNV. However further research is necessary in order to draw general conclusions regarding the role of ticks in the introduction of WNV to new areas and as virus reservoir and bridge-vector.

## Conclusions

Infected ticks on migrating birds may carry (new) pathogens to other areas much more efficiently than their avian hosts. The determination of the complete sequence of the currently in Romania circulating WNV strain revealed the most similar genetic relationship to the neuroinvasive Russian WNV strain Reb_Volgograd_07_H. Based on these sequences, future evolution of the Eastern European lineage 2 WNV cluster may be monitored.
